# Preparation of recombinant glycoprotein B (gB) of Chelonid herpesvirus 5 (ChHV5) for antibody production and its application for infection detection in sea turtles

**DOI:** 10.1038/s41598-022-15281-9

**Published:** 2022-06-30

**Authors:** Tsung-Hsien Li, Wei-Li Hsu, Chang-You Chen, Yi-Chen Chen, Yu-Chen Wang, Ming-An Tsai, I.-Chun Chen, Chao-Chin Chang

**Affiliations:** 1 National Museum of Marine Biology & Aquarium, Checheng, Pingtung 94450 Taiwan; 2 Department of Marine Biotechnology and Resources, National Sun Yat-Sen University, Kaohsiung, 80424 Taiwan; 3 Institute of Marine Ecology and Conservation, National Sun Yat-Sen University, Kaohsiung, 80424 Taiwan; 4 Graduate Institute of Microbiology and Public Health, College of Veterinary Medicine, National Chung Hsing University, Taichung, 40227 Taiwan; 5 Division of Endocrinology and Metabolism, Department of Internal Medicine, Taichung Veterans General Hospital, 1650 Sec. 4 Taiwan Blvd., Xitun Dist., Taichung, 407 Taiwan; 6 Department of Veterinary Medicine, College of Veterinary Medicine, National Pingtung University of Science and Technology, Pingtung, 912301 Taiwan; 7 International Program in Ornamental Fish Technology and Aquatic Animal Health, National Pingtung University of Science and Technology, Pingtung, 912301 Taiwan

**Keywords:** Ecology, Immunology, Zoology, Diseases, Pathogenesis

## Abstract

The Chelonid herpesvirus 5 (ChHV5) infection possibly associated to the fibropapillomatosis (FP) disease in sea turtles worldwide remains largely unknown and limited studies have used serological approaches to detection of antibodies against ChHV5 in sea turtles with or without FP. We aimed to develop diagnostic platforms based on the viral glycoprotein B (gB) for ChHV5 infection. In this study, five recombinant sub-fragments of the gB protein were successfully expressed and subsequently served as antigens for both seroprevalence and antibody production. The results indicated that the five expressed proteins harbored antigenicity, shown by the results of using sera from sea turtles that were PCR-positive for ChHV5. Moreover, seropositive sea turtles were significantly associated with FP (p < 0.05). We further used the expressed protein to produce antibodies for immunohistochemical analysis, and found that the in-house-generated sera specifically stained FP lesions while normal epithelium tissues remained negative. Of major importance, the reactivity in the ballooning degeneration area was much stronger than that in other regions of the FP lesion/tumour, thus indicating ChHV5 viral activities. In summary, the developed serological test and specific anti-gB antibodies for IHC analysis could be applied for further understanding of epidemiological distributions of ChHV5 infection in sea turtles, and studies of ChHV5 pathogenesis.

## Introduction

Fibropapillomatosis (FP) is a tumor-forming disease distributed globally in sea turtles^1^. In sea turtles with FP, tumors can appear on the eyes, mouth, skin and even internal organs, including the heart, lung and kidney^2,3^. Among the seven species of marine turtles in the world, green turtles appear to be severely affected by FP^1^ and reports exist for even asymptomatic animals infected; unlike productive infection, herpesviruses also establish latency status which presents no evidence of clinical signs and a lower level of viral DNA^4^. Fibropapillomatosis is frequently observed in immature green turtles and less commonly reported in adults^5,6^. Severe FP in sea turtles may lead to immunosuppression, poor body condition and lower survival rates^7–9^. It has been hypothesized that FP could be associated with environmental factors or infectious agents, among which Chelonid herpesvirus 5 (ChHV5) is a presumed etiological agent of FP^5,10^.

Chelonid herpesvirus 5 (ChHV5) is an enveloped, double-stranded DNA virus. According to the current taxonomic classification, ChHV5 has been placed in the family Herpesviridae, subfamily Alphaherpesvirinae, genus *Scutavirus*^5,11^. Early attempts at culturing ChHV5 in vitro have not yet succeeded^12,13^. Recently, ChHV5 was successfully isolated using organotypic skin cultures^14^. The results also indicated that ChHV5 may play a significant role as the cause of FP in sea turtles. However, the presence of ChHV5 does not always result in FP formation and is found in many turtles that never show any sign of FP disease^15,16^. The transmission route of ChHV5 among sea turtles is still unknown, possibly through direct contact^17–20^. Several reports also suggested that the transmission route of ChHV5 could occur via body fluids^21,22^. Transmission of ChHV5 has also been demonstrated through the water column, potentially via leech and fish vectors, and potentially vertically from mother to offspring^23–26^. A previous study^20^ that collected FP tumor samples from sea turtles (*Chelonia mydas*) found that only 7% of tumors had inclusion bodies within the epidermis while 65% of sea turtles presented no inclusion bodies in FP tumors. Tumor volume has also been found to be inversely proportional to the quantity of inclusion bodies^20^. Therefore, Kemper^27^ inferred that this phenomenon may be associated with superspreaders. In other words, it is possible that only a small number of sea turtles with FP are able to spread the infection, which means that these superspreader turtles play a crucial role in spreading the disease throughout the population^20^.

It is important to develop diagnostic tools that can be applied to understand the epidemiology of ChHV5 infection in sea turtles. Currently, studies that use histologic sections from tumors to identify viral inclusion bodies and/or perform ChHV5 detection by PCR may underestimate the infection prevalence^20,22,28^, and therefore it has been shown that using a triplet set of singleplex PCR outperforms other methods by threefold increase in detection^4^. Furthermore, performing tissue biopsies on sea turtle tumors would substantially increase the cost of diagnostic tests and have low diagnostic sensitivity. Tumors at an early stage may also be difficult to observe by gross pathological examination.

As a consequence of the aforementioned circumstances, the development of serological approaches for detection of antibodies to ChHV5 has been previously described. For example, Herbst et al. used immunohistochemistry assay to evaluate the antibody reactivity to herpesvirus inclusions in FP tumor tissues in Florida green turtles from habitats where FP is enzootic and habitats free of FP. The study found that turtles from areas where heavily affected by FP were significantly more likely to have antibodies to herpesvirus inclusions than turtles from FP-free areas^29^. Subsequently, Herbst et al. expressed glycoprotein H from ChHV5 in a baculovirus system and designed an indirect ELISA test to detect the antibodies reactivity of tumored and non-tumored Floridian sea turtles to ChHV5. Nonetheless, they observed a seroconversion rate from turtles that had experimentally induced FP tumors was only 50%, whereas turtles from wild had an approximately 80–100% seroprevalence, regardless of FP disease status or whether or not turtles originated from FP-free areas or areas affected by FP. Therefore. Herbst et al.^30^ further concluded either that sea turtles with ChHV5 infection is widespread in Florida or that there is antibody cross-reactivity between ChHV5 and other herpesviruses. In addition, Work et al.^31^ established an ELISA test using two expressed ChHV5 glycoprotein antigens to detect antibodies against the ChHV5 in the serum of sea turtles from Hawaii and Florida, and compared their differences in antibody responses. They observed a seropositive to ChHV5 was uniformly in green turtles from Florida regardless of FP tumor status. However, regarding the turtles from Hawaii, the levels of antibody response to ChHV5 were higher in turtles with FP than in turtles without FP.

Therefore, the aim of this study was to develop potential alternative methods for the diagnosis of ChHV5-infected sea turtles. One method produces ChHV5 glycoprotein B (gB), the dominant immunogen, in a prokaryotic expression system for serological diagnosis, and another method generates gB-specific antisera by using purified recombinant proteins for ChHV5 detection. The developed methods could be applied in the field to provide a better scientific understanding of the epidemiological distributions of ChHV5 infection in sea turtles. Prospectively, the information may improve the management of these animals at the early stage of infection.

## Materials and methods

### Sample collection from sea turtles

In total, 45 serum samples from 33 juvenile green turtles (*C. mydas*), including 6 sea turtles with tumors, 5 juvenile hawksbill turtles (*Eretmochelys imbricate*), and 7 olive ridley turtles (*Lepidochelys olivacea*) (juvenile = 5; sub-adult = 2). All turtles were sourced from: eastern Taiwan (n = 24), southern Taiwan (n = 14), central Taiwan (n = 6), and northern Taiwan (n = 1). Among the 45 sea turtle samples, 6 green turtles developed FP (n = 1 with tumor score 1; n = 1 with tumor score 2; n = 4 with tumor score 3)^32^, while 39 did not have FP. FP tumor tissues were collected from 6 green turtles (from shoulder/flippers/inguinal regions) with FP during surgical procedures. Regarding the collection of normal skin tissues, one normal skin tissue (from shoulder) was collected from one necropsied dead green turtles (stranding and discovered from southern Taiwan) confirmed without FP. All tissue samples were fixed in 10% neutral buffered formalin prior to further analysis. In this study, all sea turtles were discovered and rescued through the official reporting system of the Marine Animal Rescue Network (established by the Ocean Conservation Administration) and admitted to the National Museum of Marine Biology and Aquarium (NMMBA), between 2017 and 2020.

### Detection of ChHV5 DNA by polymerase chain reaction (PCR)

Total DNA was extracted from blood of 45 sea turtles by DNeasy blood & tissue kit (Cat. No. 69504, Qiagen, Valencia, CA, USA) following manufacturer's instructions. Subsequently, the ChHV5 infection status all 45 sea turtles was determined by PCR using primers targeting on UL18 (capsid protein gene), UL22 (glycoprotein H gene), and UL27 (glycoprotein B gene) regions^4^. The sequence of primer sets are: UL18F: 5′-CACCACGAGGGGGAAAATGA, UL18R:5′-TCAAATCCCCCGTTCACTCG; UL22F: 5′-ACGGCGTTGGCTAGTGAATC, UL22R: 5′-GCAGTTCGGTACACACCTCT; UL27F: 5′-TAACAAGAAAGAACCGCGCG; UL27R: 5′-ATTTTCCCGGTCAGTGCCAA. PCR amplifications were performed in a total volume of 50 μl. The reaction included 1 μl of the template DNA, 1 μl of each primer (10 μM), 22 μl of distilled water (DDW), and 25 μl of the AmpliTaq Gold^®^ 360 Master Mix (Cat. No. 4398876, Life Technologies, Valencia, CA, USA). The thermocycle for amplification was: Initial denaturing at 95 °C for 10 min, followed by 40 cycles of 95 °C for 30 s, 55 °C for 30 s, and 72 °C for 60 s, and then a final extension at 72 °C for 7 min. Results were visualized by gel electrophoresis (2% agarose) with SYBR Safe DNA Gel Stain (Cat. No. S33102, Invitrogen, Carlsbad, CA, USA).

### Sequence optimization of the UL27 gene for expression of the ChHV5 glycoprotein protein using *E. coli*

To express large quantities of ChHV5 gB, we adopted the prokaryotic *Escherichia coli* (*E. coli*) expression system. The construct (namely UL27/pUC57) containing sequences of the full length UL27 fused with FLAG tag sequence (GenBank accession no. AF035003.3) was synthesized by Allbio Science Co., Ltd, Taiwan. The sequence information of the glycoprotein (gB) datasets used and analyzed for protein expression during the current study was obtained and available from the GenBank repository [https://www.ncbi.nlm.nih.gov/nuccore/AF035003.3]. Considering the difference in tRNA-codon usages between prokaryotes and eukaryotes would possibly affect subsequent protein expression, the optimized UL27 gene sequence, without altering the translated amino acid sequences, to fit the *E. coli* expression system was synthesized. The codon optimized UL27 gene was further used as the template for amplification of different gene fragments by Polymerase Chain Reaction (PCR).

### Construction of plasmids expressing partial fragments of ChHV5 gB protein

To determine the relative antigenicity and also to increase the expression yield, plasmids expressing various regions of gB protein were constructed. Briefly, the five regions covering different fragments of the UL27 gene were amplified from plasmid UL27/pUC57 by PCR using specific primer sets with built-in restriction enzyme sequences shown as underlined in Table 1. The thermal cycling conditions were: 98 °C (5 min) followed by 35 cycles of denaturation (98 °C, 30 s), annealing (58 °C, 1 min), and extension (72 °C, 2 min), and finished with a final extension (72 °C, 10 min). PCR amplicons with expected sizes were isolated from gel and trimmed with the restriction enzymes followed by ligation with vectors either pET24a or pET32b (Novagen, Germany) linearized with the same restriction enzymes. The resulting plasmids with expected insert sizes as confirmed by restriction enzymes were sent for automated DNA sequencing (Mission Biotech, Taipei, Taiwan).

**Table 1:** Information on the constructs expressing the UL27 fragments. The bold characters indicate sequences recognized by restriction enzymes for the ease of further cloning procedure.

UL27 fragment	vector	UL27 primer	Primer sequences (5′ → 3′ end)	Molecular weight (kDa)	Amino acid	Sizes of PCR amplicon (bp)
F1	pET24a	Nde l-F-1	atata**catatg**attatcgtgctcgagctg	13	1–104	318
		Xho l-R-1	cgg**ctcgag**acgcacgatggtaacttc			
F2	pET24a	Nde l-F-2	cgcg**catatg**agctaccagacctttctg	12.7	105–203	303
		Xho l-R-2	g**ctcgag**gttcacggtcacgctctc			
F3	pET24a	Nde l-F-3	ccg**catatg**tgtttagtggtggacaccgtg	12.9	204–305	315
		Xho l-R-3	cgc**ctcgag**ggtttcgggcacggtctg			
F1–2	pET32b	BamHl-F-1	cgcg**ggatcc**cgagaaacgtaatttaactttaagc	40.1	1–203	621
		Xho l-R-2	g**ctcgag**gttcacggtcacgctctc			
F2–3	pET24a	Nde l-F-2	cgcg**catatg**agctaccagacctttctg	24.4	105–305	618
		Xho l-R-3	cgc**ctcgag**ggtttcgggcacggtctg			

### Expression of recombinant gB fragments in *E. coli*

In the current study, the recombinant gB protein is a key reagent that served as antigen for seroprevalence of ChHV5 as well as for the generation of ChHV5 gB antibody (conducted by Yao-Hong Biotech Inc., Taiwan). The plasmids expressing individual gB fragment were transformed into *E. coli* host cells, strain BL21 (DE3), Rosetta. Expression of all the recombinant gB fragments was induced by 0.8 mM of isopropyl β-d-1-thiogalactopyranoside (IPTG) at 28 °C for 16 h. As all the gB fragments cloned into the pET series vectors were expressed as a fusion protein with a 6-histidine tag at C-terminus end, they could be further purified by Ni–NTA column chromatography using the chelating Sepharose Fast Flow (GE Healthcare) following the method described in one previous study^33^. The yield and purity of recombinant gB proteins were confirmed by sodium dodecyl sulfate polyacrylamide gel electrophoresis (SDS-PAGE). Subsequently, 6 M urea and 0.4 M imidazole contained in the purified protein were depleted by step-wise dialysis against 1 × PBS buffer (0.02 M phosphate, 0.15 M NaCl) with gradually decreased concentrations of urea at 4 °C. The concentration of recombinant proteins were then estimated by National Institutes of Health ImageJ software (https://imagej.nih.gov/ij/, 1997–2018.) using the standard curve established by bovine serum albumin (BSA) with known concentrations^42^.

### Western blot analysis

Recombinant gB fragments were separated by 12.5% or 15% SDS-PAGE and electrotransferred to PVDF membrane by using Mini Proten III apparatus (Cat. No. 165-3301, BioRad). The filters were blocked in PBS-T buffer (0.02 M phosphate, 0.15 M NaCl, 0.05% Tween-20) containing 5% skimmed milk and reacted with mouse anti-his tag antibody (1:5,000, Cat. No. GTX40628, GeneTex) at 4 °C for overnight. After six-time wash with PBS-T buffer, the PVDF filter was then incubated with the secondary antibody, 1:5000 diluted goat anti-mouse IgG conjugated with horseradish peroxidase (HRP), or 1:500 diluted Protein A/G-HRP (Cat. No. 32400, Thermo fisher scientific™, United States) for sea turtle antibody detection, at room temperature for 1 h followed by PBS-T wash to remove the unbound antibodies. Ultimately, the signal was detected by ECL reagents (Thermo Fisher Scientific, United States) and the image was acquired by ImageQuant LAS 4000 Mini (GE Healthcare).

### Immunohistochemical (IHC) analysis

To establish IHC protocol, normal skin tissue from PCR-negative sea turtles served as the negative control. In total, the FP on skin tissue from six individual sea turtles that were detected positive for ChHV5 DNA (positive tissue samples), and one normal tissue detected negative (the negative tissue) were included in the IHC analysis.

IHC procedure was conducted as reported in our previous study^34^. In brief, sections of formalin-fixed and wax-embedded skin tissues of sea turtles were made using a rotary microtome (Leica RM2245, Leica Biosystems, Germany) and were further deparaffinized and rehydrated. Antigen retrieval was carried out by heat-induced epitope retrieval method: slides immersed into boiled sodium citrate buffer (0.01 M, pH 6.0), which was preheated up to 100 °C, for 20 min and cooled at room temperature for 20 min. Subsequently, the slides were incubated with peroxidase-blocking reagent (Cat. No. S200389, Dako, Denmark) for 30 min, and then treated with or without primary antibodies (the anti-gB serum prepared from this study). In each interval of the following procedures, sections were rinsed with a mixture of TBST buffer. Tissue sections were then reacted with secondary antibody (HRP anti-rabbit/mouse, DAKO, Denmark), followed by incubation of DAB and chromogen (dilution 1 μL in 100 μL) from a commercial ChemMate EnVision detection kit (Cat. No. K5007, Dako, Denmark). Ultimately, tissue sections were counterstained with Mayer’s hematoxylin reagents (Code S3309, Dako, Denmark) for 2 min followed by wash with DDW, and reacted with 37 mM ammonia water for 5 s and rinsed with DDW.

### Immunofluorescent assay (IFA)

Human 293 T cells were transfected with plasmids expressing full-length ChHV5 gB protein fused with FLAG tag at its C-terminus. At 24 h post transfection, 293 T cells (CRL-3216, ATCC, USA) were fixed with 2% formaldehyde for 10 min, followed by permeabilization with 0.1% Triton X-100 for another 10 min. Subsequently, cells were incubated with anti-FLAG antibody (1:500) (F7425; Sigma-Aldrich), or antisera (F1, F2, F3, F2–3) at the dilution of 1:500 for 1 h at room temperature. After six times of washes with PBS containing1% bovine serum, goat anti-mouse IgG (1:2,000 fold diluted) (Cat. No. A28175, Alexa Fluor® 488, Invitrogen) was used as secondary antibody. After one-hour incubation, nuclei were stained with 4, 6-diamidino-2-phenylindole (DAPI, Cat. No. D9542, Sigma-Aldrich) for 10 min, followed by confocal microscopy (FV1000, Olympus, Tokyo, Japan) with Olympus FV10-ASW 1.3 viewer software.

### Statistical analysis

To evaluate the association between seropositivity and FP or viremia tested by PCR of UL27 gene, Fisher’s exact test was applied due to very limited number of sea turtles with FP. The statistical significance was determined by p < 0.05. The software R (version 4.0.1) was used for statistical computing.

### Ethics statement

No sea turtles were specifically captured for the purpose of this study. All experiments were performed in accordance with relevant guidelines and regulations, and reported in accordance with the ARRIVE guidelines. The scientific permit numbers for research were obtained from the Institutional Animal Care and Use Committee (IACUC) of the NMMBA (Permit no. 2017005, 2018007, and 2020001), the Forestry Bureau (Permit no. 1071655703), and the Ocean Conservation Administration (Permit no. 1070003700 and 1080007820).

## Results

### Production of antigenic gB fragments in *E. coli*

**Figure 1:**
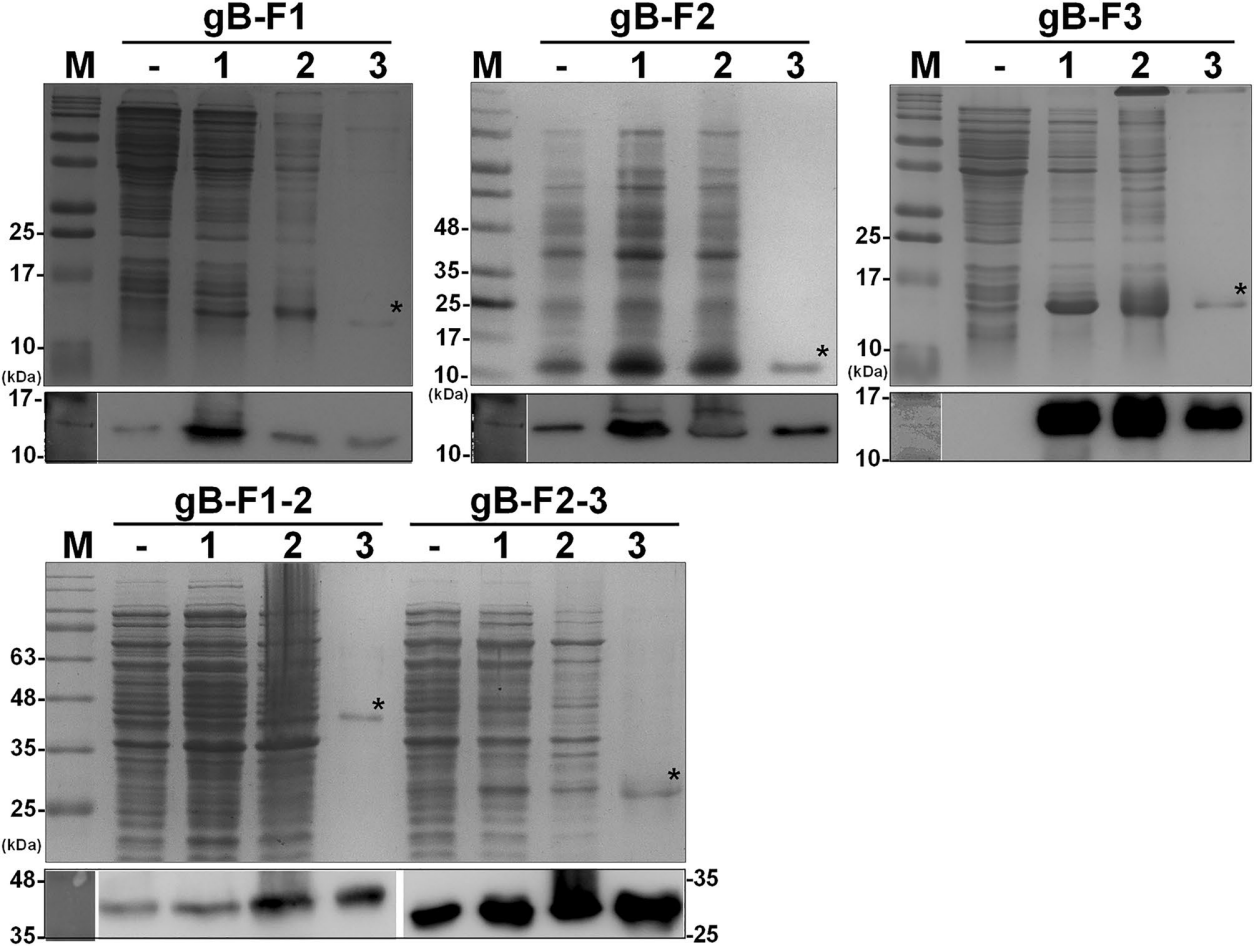
Generation of recombinant partial gB proteins. Five antigenic fragments of gB protein, labeled gB-F1, gB-F2, gB-F3, gB-F1–2 and gB-F2–3, were expressed in *E. coli* under IPTG treatment (Lanes 1, 2, 3) and purified by Ni–NTA affinity chromatography. Purified proteins (Lane 3) with the expected molecular weight are indicated as asterisks. Five gB fragments were expressed as a fusion protein with a six-histine tag at its C-terminus, which was confirmed by western blot analysis with an anti-histidine antibody (shown in the panel below SDS-PAGE). Line—indicates the mock control lysate without IPTG induction, and Lanes 1 and 2 are the total and insoluble fractions of bacterial lysate, respectively. (For gB-F1, gB-F2 and gB-F3, not the whole blot was used to react with antibody. Due to the molecular weights of target proteins gB-F1, gB-F2 and gB-F3 are very small (around 10 kDa), the protein-transferred membrane was trimmed to the region between 15 and 10 kDa below for further antibody hybridization).

Diagnostic reagents for ChHV5 are not commercially available; therefore, the initial goal of the current study was the development of ChHV5-specific immunodiagnostic systems. Initially, an attempt was made to produce the full-length ChHV5 gB protein. Due to the low yield of gB expression, six partial gB fragments were further produced to study the ChHV5 seroprevalence and determine the relative antigenicity of the gB protein. Referring to the Kyte and Doolittle hydrophilicity plot analysis^35^, the sequences of UL 27 encoding the gB protein were divided into six fragments (containing 100–150 amino acids of each) and cloned into the pET24a vector for protein expression. The six gB fragments F1–F6 contain regions spanning 1–104, 105–203, 204–305, 306–477, 478–746, and 747–853 residues, with molecular sizes of approximately 13 kDa, 12.7 kDa, 12.9 kDa, 20.5 kDa, 30.7 kDa, and 13.2 kDa, respectively. Among the six recombinant partial gB proteins, only F1, F2, and F3 of N-terminal gB were successfully expressed (Supplementary Fig. 1). Hence, two additional constructs, namely, F1–2 and F2–3 expressing 1–203 and 105–305 residues, were produced to generate fragments covering a longer range of gB proteins. Of note, considering the available options of resection enzymes for cloning, a fragment of F1–2 was cloned into the pET32b vector and expressed as a fusion protein, with thioredoxin at its C-terminus. As indicated in Fig. 1, recombinant gB fragments with expected sizes were expressed at a higher level (Lane 1). Of note, fractionation analysis indicated that all the gB fragments remained as insoluble forms (Fig. 1, Lane 2), although low-temperature conditions (i.e., 20 °C, 25 °C, 28 °C) was applied for the cultivation of *E. coli*. The gB fragments were further purified (Fig. 1, Lane 3).

### Detection of ChHV5 antibodies using purified recombinant proteins

**Figure 2:**
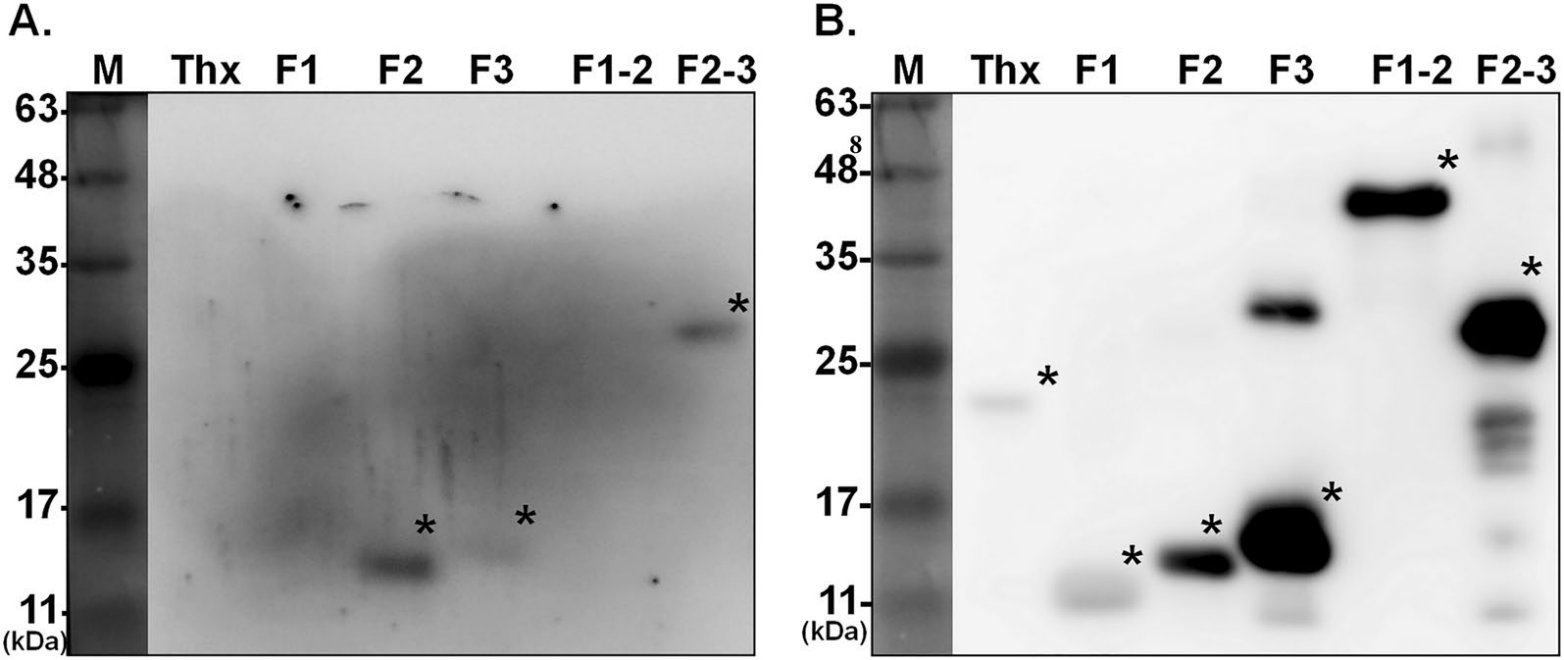
Immunoblot analysis of the antigenicity of recombinant gB proteins in the serum of sea turtles. Five antigenic fragments of gB protein and the fusion tag protein thioredoxin (Thx), which serves as a negative protein for turtle serum, were reacted with either sea turtle serum (**A**) or the positive control anti-His antibody (**B**). The gB fragments recognized by the tested sera are indicated with asterisks.

Next, the five purified recombinant proteins serving as antigens were used to determine the ChHV5 infection status of sea turtles by immunoblot analysis. As shown in Fig. 2, five gB fragments can be recognized by anti-histidine antibody (Panel B). Notably, the F2, F3 and F2–3 gB fragments reacted with the serum of one FP-affected sea turtle that was infected by ChHV5 based on PCR assay (Fig. 2A).

Subsequently, the seropositivity of ChHV5 in the 45 sea turtle serum samples was determined, and the possible association of ChHV5 infection with FP in sea turtles was further evaluated. Among the 45 sea turtle samples, 6 green turtles developed FP, while 39 did not bearing FP. The results indicated that the serum samples collected from sea turtles with FP showed seropositivity to recombinant proteins of F1, F2, F3, and F2-3, and the seropositivity percentages were 67%, 100%, 100%, and 100%, respectively (Table 2). Furthermore, an FP-positive status was significantly associated with seropositivity (p < 0.05; tested through Fisher's exact test), especially when using F3 and F2–3 as the antigens, although this association was not observed when F1–2 was used as the antigen. Moreover, attempt was made to investigate the association of seropositivity and PCR positivity. In total, 37 turtles were detected positive with ChHV5 by PCR, while 8 samples were negative. However, when using PCR results to determine ChHV5 infection, an association was not observed between PCR positivity and seropositivity, regardless of how we applied the various recombinant proteins for seropositivity determination.

**Table 2:** Antibody responses were distinct between the sea turtles with and without fibropapillomatosis according to the recognition of the recombinant ChHV5 gB protein.

Protein	FP disease status		p-value^b^	Viremia status^a^		p-value^b^
	Turtles with FP tumours (n = 6 ^c^)	Turtles without FP tumours (n = 39)		Positive (n = 37)	Negative (n = 8)	
Thioredoxin	0	0	NA	0	0	NA
Fragment 1	67%	21%	0.04	30%	13%	0.42
Fragment 2	100%	44%	0.02	57%	25%	0.13
Fragment 3	100%	28%	0.002	43%	13%	0.13
Fragment 1–2	0%	5%	1	2%	13%	0.33
Fragment 2–3	100%	28%	0.002	43%	13%	0.13

^a^Determined by PCR using UL18, UL22 or UL27 specific primers. Any of the results showed positive were considered as PCR positive ones and with viremia status.

^b^By fisher’s exact test.

^c^All green turtles.

To further understand whether the association of ChHV5 infection and seropositivity could be affected by the species of sea turtles, a stratified analysis based on sea turtle species was conducted. Because study subjects were limited among hawksbill sea turtles (5 turtles) and olive ridley sea turtles (7 turtles), only samples from green sea turtles were analyzed. The results indicated that 6 green turtles with FP were only significantly associated with seropositivity when using F3 and F2-3 as antigens (p < 0.05; tested through Fisher's exact test). Although an association between PCR positivity of ChHV5 and seropositivity was not detected by F3 and F2–3 (p > 0.05; tested through Fisher's exact test), a higher percentage of seropositivity were found in PCR-positive green turtles (F3 = 59%; F2–3 = 59%) than in PCR-negative green turtles (F3 = 16%; F2–3 = 16%).

### Evaluation of the sensitivity and specificity of the developed serological test for ChHV5 infection

The results indicated that green turtles with FP were only significantly associated with seropositivity when using F3 and F2–3 as antigens; therefore, we further evaluated the sensitivity and specificity of the developed serological tests using F3 or F2–3 as antigens. We checked whether seropositivity detected by the F3 and F2–3 antigens was correlated with the FP of sea turtles and compared the correlations between the PCR analysis results and serologic method results based on the two aforementioned antigens (Table 2). The results showed that when the presence of FP was considered the gold standard for ChHV5 infection, the sensitivity and specificity of the developed serological test were 100% (6/6) and 71.8% (28/39), respectively. However, when sea turtles with either FP or positive PCR results were considered the gold standard for ChHV5 infection, the sensitivity and specificity of antibody detection were 43.2% (16/37) and 87.5% (7/8), respectively (Table 2).

### Establishment of immunohistochemical staining (IHC) for detection of ChHV5 gB protein in tumors from sea turtles

**Figure 3:**
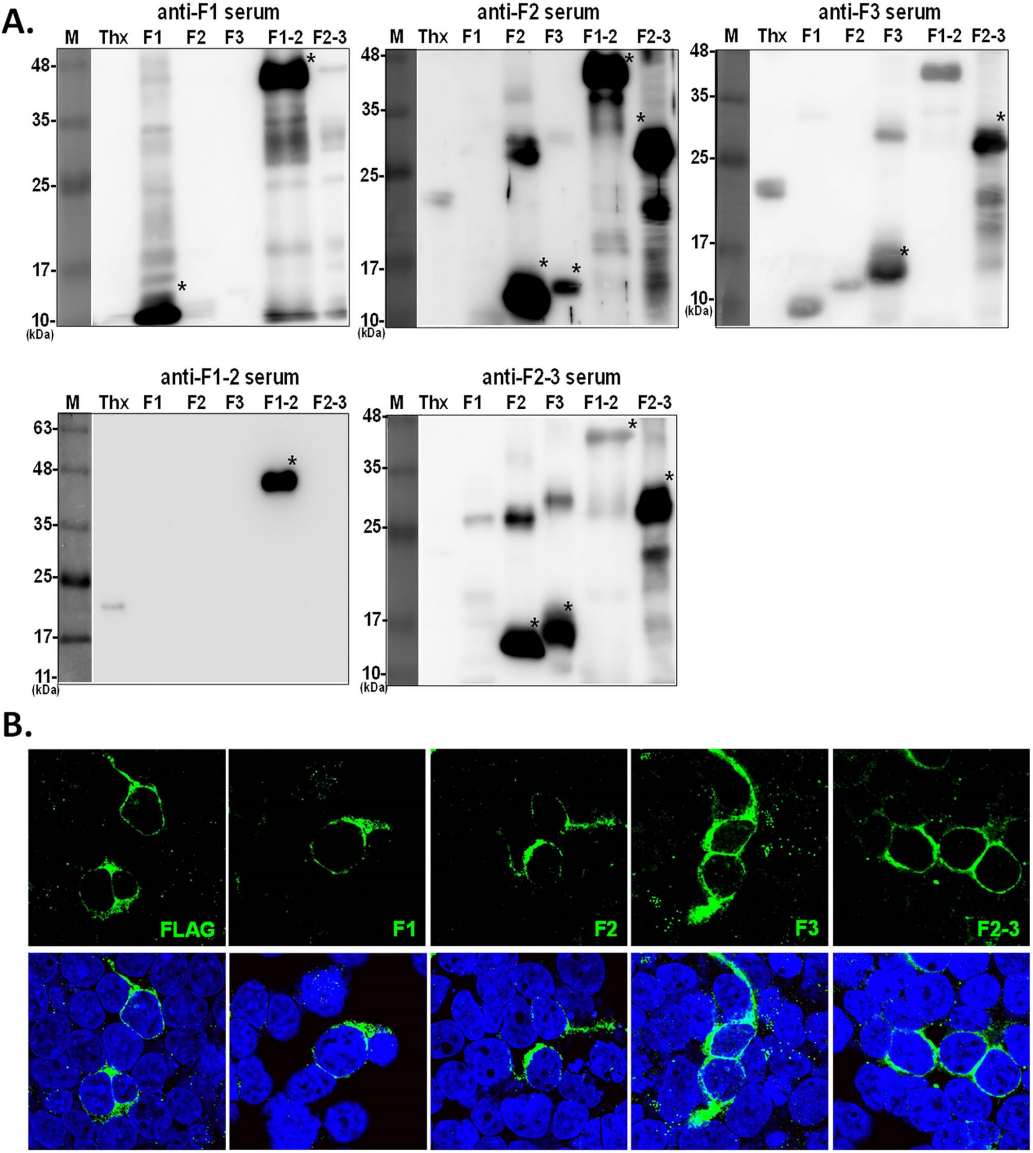
Verification of the specificity of anti-serum samples from mice immunized with various recombinant gB proteins. (**A**) Five antigenic fragments of gB protein and the fusion tag protein thioredoxin (Thx) were reacted with serum samples collected from mice immunized with F1, F2, F3, F1–2, and F2–3, as labeled on the top of each blot. The partial gB proteins specifically recognized by the mouse sera are indicated with asterisks. (**B**) Human 293 cells transiently expressing the full-length gB protein fused with a FLAG tag were used to confirm the specificity of antiserum F1, F2, F3, and F2–3. FLAG served as a positive control antibody against the gB-FLAG fusion protein. By IFA, the green fluorescence located in the cytoplasm indicated positive staining of gB. The nucleus was shown in blue by DAPI staining.

To study the relationship of ChHV5 infection with FP formation, we developed an IHC platform. Initially, the five recombinant gB fragments were used to yield anti-serum in rabbits or mice, and the results were validated by western blot analysis. The results indicated that all five antisera could specifically recognize the corresponding recombinant protein fragments, as indicated by asterisk signs in Fig. 3A. Of note, because of the presence of the communal histidine tag at the C-terminus of target proteins, antisera anti-F3, anti-F2–3 and anti-F1–2 detected the recombinant gB proteins and cross-reacted with the control protein, namely, the thioredoxin-histidine fusion protein (Fig. 3A). In addition to verification by western blot assay, the antisera with strong reactivity were further tested by immunofluorescence assay (IFA). As shown in Fig. 3B, antiserum F1, F2, F3, and F2–3 recognized the full-length gB protein transiently expressed in human 293 cells, thus ensuring that the antisera can recognize the gB protein in both denatured and native forms.

**Figure 4:**
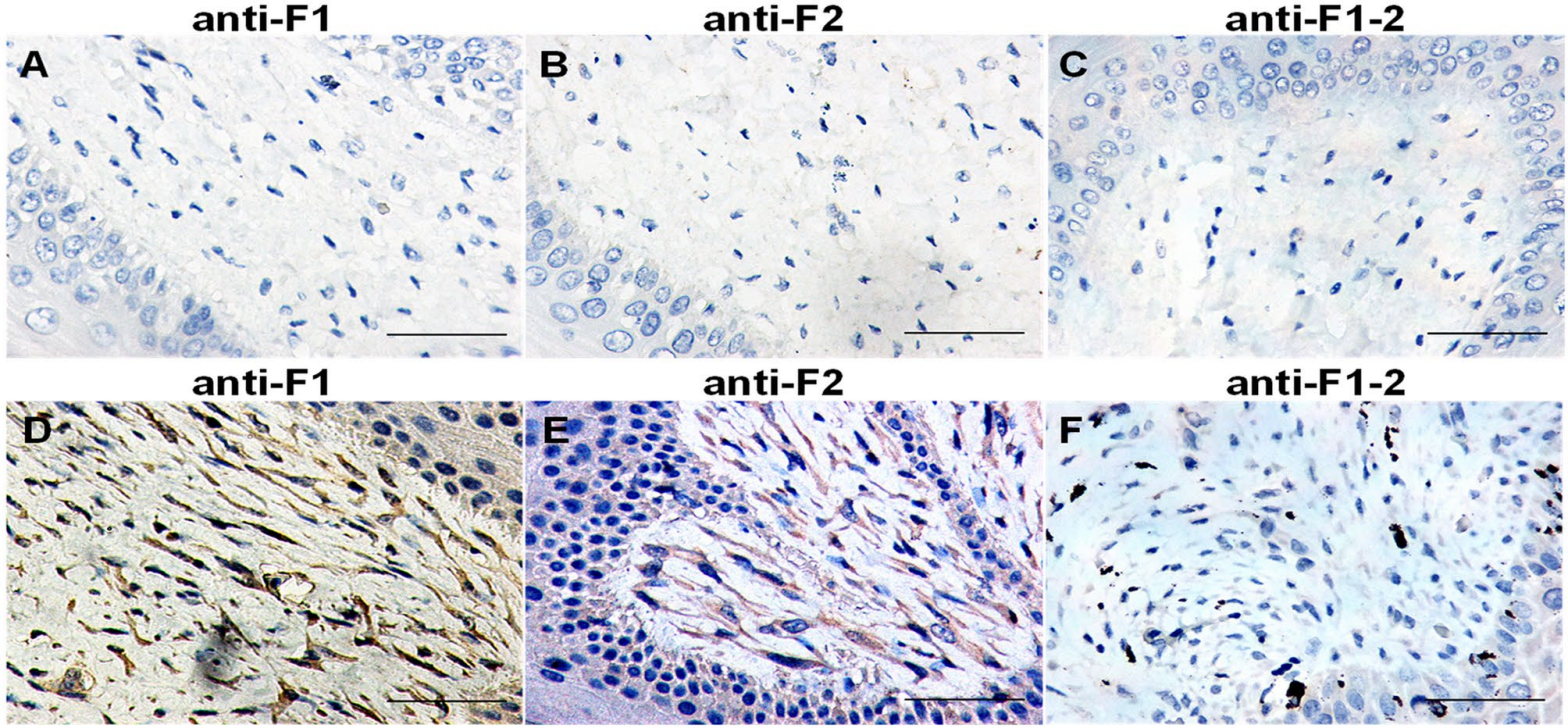
Reactivity of anti-sera from mice immunized with various recombinant gB proteins in sea turtle tissue by immunohistochemistry assay (IHC). Mouse anti-sera specific for ChHV5 gB (anti-F1, anti-F2, and F1–2) were reacted with tissue sections from healthy turtle skin samples (Panels **A**,**B**,**C**) or skin tissue with FP (Panels **D**,**E**,**F**). The scale bar in each image indicated 100 μm.

Subsequently, an IHC protocol for ChHV5 detection was optimized using antisera. Among the five antisera, the anti-F1, anti-F2 or anti-F1–2 serum did not react with healthy skin (Fig. 4A–C), while substantial positive reactivity was revealed in the nucleus of epidermal cells of FP tissues (Fig. 4D–F), indicating the expected specificity. The F2 serum showed better immunoreactivity and high specificity between healthy and FP tissue and was then selected for optimization of IHC.

**Figure 5:**
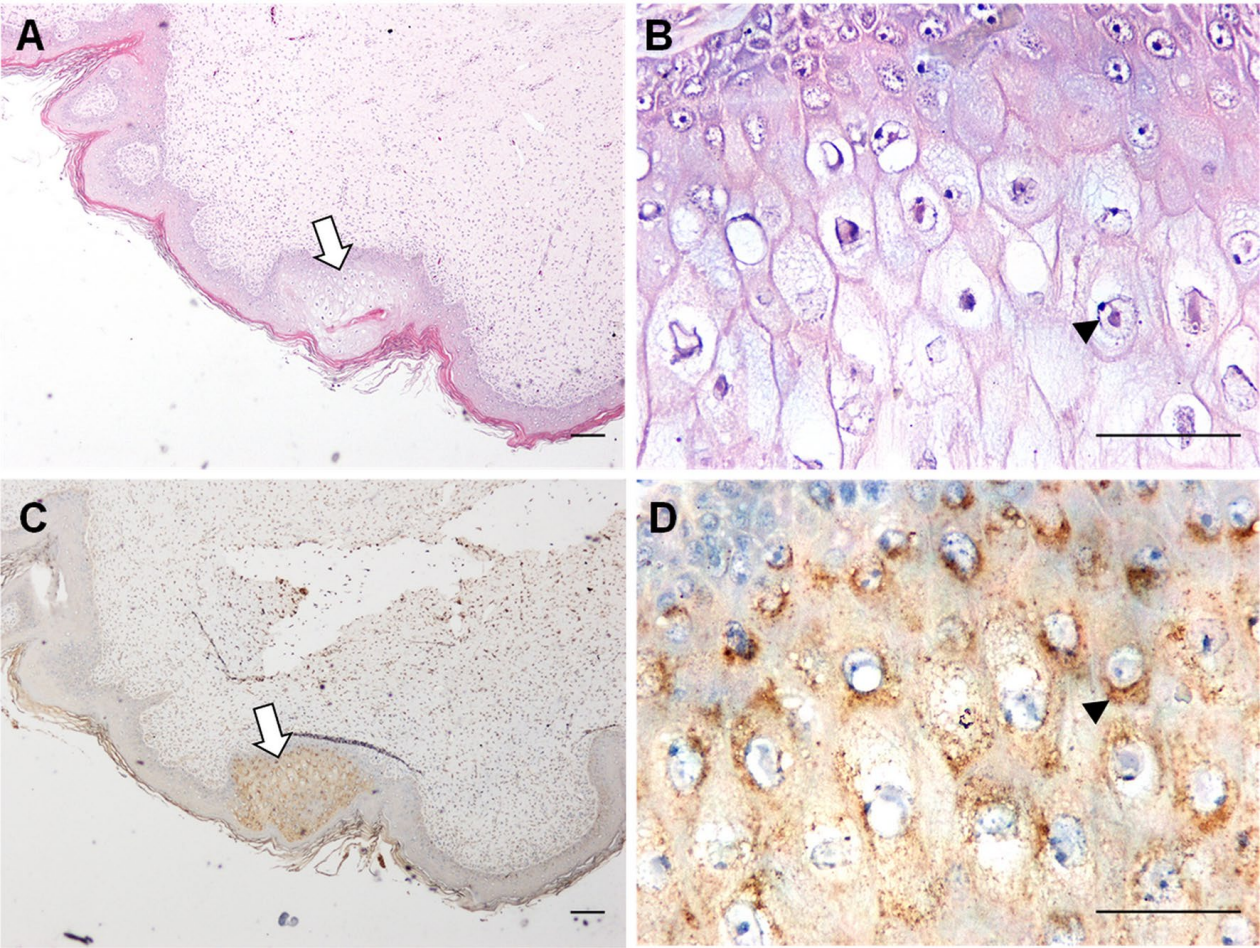
Reactivity of anti-serum F2 with FP-bearing sea turtle skin by IHC. Skin tissue sections derived from sea turtles (G46) with FP were stained with hematoxylin–eosin reagent (Panels **A**,**B**) or with mouse anti-sera F2 (Panels **C**,**D**). The white arrow and black arrowhead indicate ballooning degeneration and inclusion bodies, respectively, in the tumor tissues. The scale bar in each image indicated 100 μm.

Two juvenile green turtles, G46 (tumor score = 2) and G57 (tumor score = 3), which were tested as positive with ChHV5 DNA and gB protein by PCR and western blot analysis, respectively, were selected for IHC analysis. As shown in Fig. 5, ballooning degeneration and eosinophilic intranuclear inclusion bodies were identified in the FP tissues of two sea turtles (No. G46 and G57) by histopathological examinations based on hematoxylin–eosin staining, as indicated by arrows and arrowheads in Fig. 5A,B, respectively. These pathological areas displayed strong positive reactivities to anti-F2 serum, thereby indicating the overexpression of ChHV5 gB proteins (Fig. 5C,D).

## Discussion

Because FP possibly caused by ChHV5 may threaten sea turtle life, understanding the epidemiology and effective management of ChHV5 infection plays an influential role in sea turtle conservation. To date, the detection of ChHV5 is usually based on molecular testing by PCR, which is the primary diagnostic modality for active infection^6,36–38^. However, the unknown viremia duration and latency of ChHV5 infection may possibly lead to false negative results by PCR. Hence, in our study, the triple PCR amplifying three genes (UL18, UL22, UL27), which increases ChHV5 detection rate by three folds compared to the standard assay^4^, was employed to ensure the viremia status of all sea turtle samples. Because vaccines are not available for herd protection of endangered sea turtle species, serological tests that measure anti-ChHV5 antibodies post-infection may be necessary for timely prevention and treatment. The gB protein on the ChHV5 envelope is a crucial epitope to bind receptors on host cells with high specificity^39^. We therefore developed serological diagnostic methods based on viral gB proteins, by which antibodies in sera and ChHV5 infection in tissue were successfully detected by immunoblot assay and IHC analysis, respectively.

In this study, considering the fast growth rate, high yield, and low cost, *E. coli* was an ideal system for the expression of gB protein. The results of this study showed that the different fragments of recombinant gB protein could serve as antigens to detect antibodies in serum samples from wild sea turtles (Fig. 2). Of note, the reactivity of turtle serum with these recombinant gB proteins was not as strong as that of the control antibody against the histidine tag (Fig. 2). It is speculated that the titer of ChHV5 antibody in the serum samples of sea turtles enrolled in this study may be relatively low that leads to lower signals than the positive control antibody. Among the five gB protein fragments, F2 is the most immunogenic protein that strongly reacts with turtle serum. Furthermore, we found that green turtles with FP were significantly associated with seropositivity status. It is worth noting that no association between PCR for ChHV5 detection and seropositivity was found, and ChHV5 antibodies were detected in PCR-negative green turtles. It is very likely that these seropositive animals could have been exhibiting the latent stage of ChHV5 infection^24^ with extremely low copy number of viral DNA that is under detection level, or recovering from previous infections. And hence, in the absence of viremia, infection status cannot be revealed by nucleic acid-based method, while the antibody elicited during ChHV5 infection could be traced by immunologic methods; both PCR detection and serological method are required to confirm the infection status of ChHV5. Moreover, immunoassays using recombinant proteins F2, F3, or F2–3 indicated that sea turtles with FP consistently presented seropositive results. These findings indicate that ChHV5 replication may occur in sea turtles during the tumor development stage and induce enough antibodies for detection, which again strengthens the concept that ChHV5 infection is associated with FP.

The purified gB fragment proteins were not only used for immunoassays but also applied to produce antibodies for the development of IHC methods used to monitor ChHV5 infection in FP. Normal skin tissue from PCR-negative sea turtles served as the negative control. Two sea turtles with FP (No. G46 and No. G47), for which ballooning degeneration lesions (implying vigorous virus replication) were identified by HE staining, were used as the positive control. Moreover, anti-F1 and anti-F2 showed specific antigen–antibody reactions in tumor tissues. Nevertheless, anti-F3, anti-mouse 3-F1–2, and anti-F2–3 cross-reacted with normal sea turtle skin tissues, which implies low specificity of these sera for IHC use. When stained with F2 antibody for IHC detection, strong positive reactions were present in the ballooning degeneration lesions as well as in the surrounding region of the inclusion bodies. Our results were similar to those found in ballooning degeneration lesions identified by Work et al.^14^. Instead of the gB protein, Work et al.^20^ defined ChHV5 infection by the presence of the capsid protein VP26, which is located in the nucleus, while the inclusion body is in the cytoplasm. Taken together, our results strongly indicate that the anti-F2 antibody can be used in IHC analysis to detect ChHV5.

This study is subject to a number of limitations. First, since sea turtles are endangered animals but not the typical experimental animal model in Taiwan, generation of standard ChHV5 antibody through artificial viral infection is impossible. Also, the ChHV5 antibody is not commercially available and therefore the verified positive and negative sea turtle serum samples were lacking. Therefore, several strategies were taken to ensure the specificity of the detection system. In this study, instead of using the control antibody (sea turtle serum), the specificity of immunoassay was indicated by differential reactivity of FP-affected sea turtle serum with different proteins (gB fragments or negative control protein). As described in the result section, serum from one FP-affected sea turtle (positive Ab control) recognized with the gB fragments (F2, F3, and F2–3, in Fig. 2A), but not with the negative control antigen (Thioredoxin), indicting serum of FP-sea turtle specifically reacted with gB fragment. Furthermore, the validation and estimation of the sensitivity and specificity of the developed serological test were performed by considering the FP status or PCR results of ChHV5 in sea turtles. And statistical analysis was also applied to investigate the correlation of those individual gB fragments with FP formation and also viremia. In this study, two *C. mydas* sea turtles without FP and with PCR-negative results were seropositive. Whether they were actually antibody-positive animals or merely relevant to false-positive results need further verification; for instance, to follow up whether FP is developed in these two sea turtles. Not all sea turtle species that confirmed with FP were including in this study, as well as the representation of other sea turtle species such as *Caretta caretta* who more commonly experience FP and ChHV5 infection than hawksbills and olive ridleys. Therefore, future studies should aim to expand this sample pool with more species and more FP-positive individuals.

This study applied various fragments of gB proteins for the development of serological tests. The truncated gB fragments might not be able to include epitopes with complicated secondary structures present in full-length gB proteins^39,40^. Moreover, linear forms of degenerated proteins, which serve as antigens, observed in the western blot analysis could not appropriately reflect the original conformation of the epitopes^41^, which might limit the recognition of certain antibodies when the developed tool is applied in field investigations. Nevertheless, as indicated in Fig. 3B, the polyclonal antisera generated by these gB fragments could detect the full-length gB proteins under native detection conditions (i.e., IFA), which indicates that recombinant proteins retain authentic antigenicity to a certain degree.

In conclusion, the developed serological test and specific anti-gB antibodies for IHC analysis could be applied to overcome the limitations of current molecular diagnostic techniques in the field. The applied method could be used to further understand the epidemiological distributions of ChHV5 infection in sea turtles and may help improve early infection management in these animals.

## Supplementary Information

Supplementary Information 1.

Supplementary Information 2.

Supplementary Information 3.

## Data Availability

The datasets used and/or analyzed in the current study are available from the corresponding author on reasonable request.
