# Supplementary material for: Preparation of recombinant glycoprotein B (gB) of Chelonid herpesvirus 5 (ChHV5) for antibody production and its application for infection detection in sea turtles
Source: Sci Rep. 2022 Jun 30;12:11022. doi: 10.1038/s41598-022-15281-9 (PMC9246996; doi:10.1038/s41598-022-15281-9)
Supplement: Supplementary file 1 — Supplementary Information 1. [file 41598_2022_15281_MOESM1_ESM.pptx]

## Slide 1
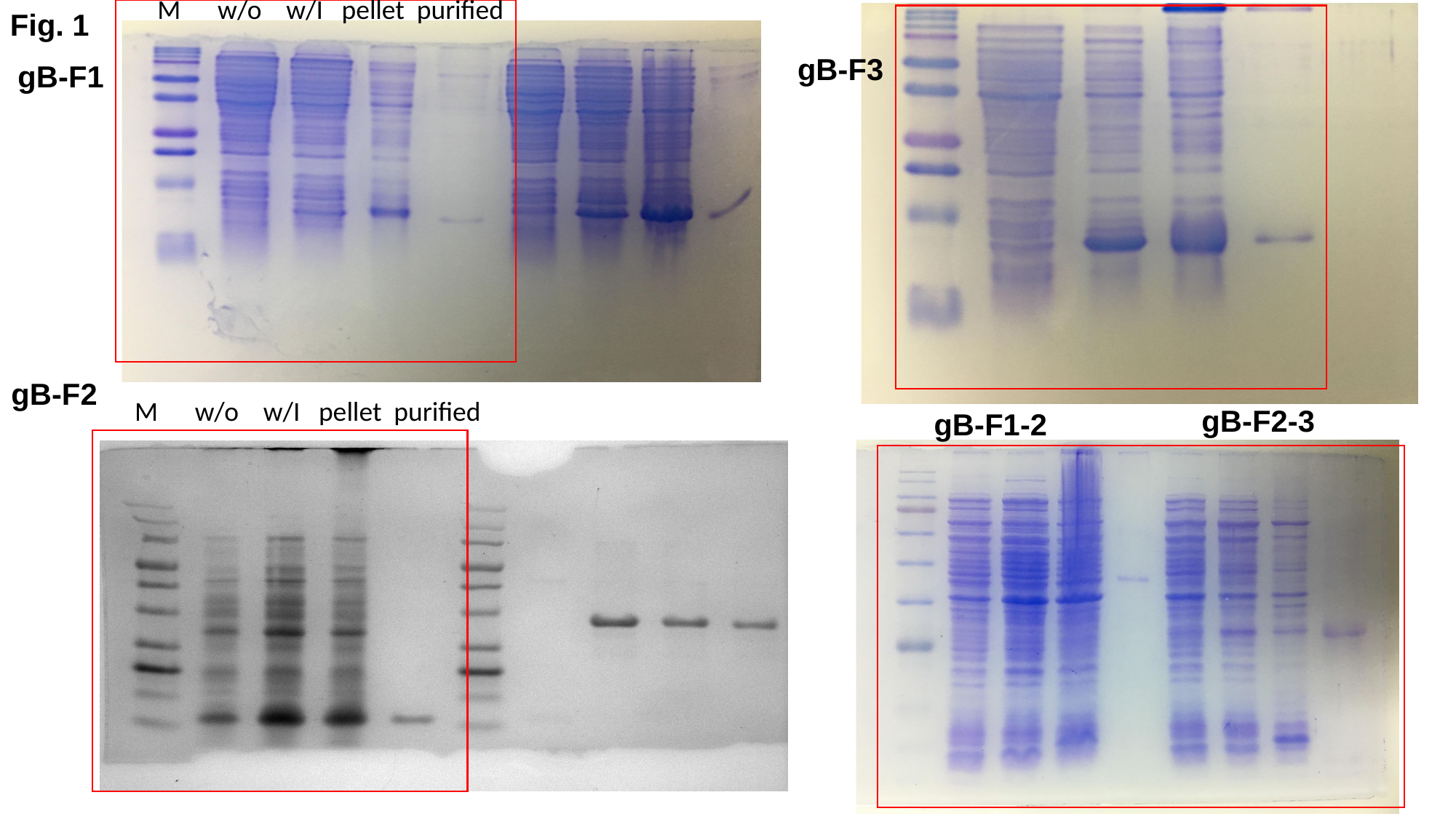

Fig. 1
M w/o w/I pellet purified
gB-F3
gB-F1
gB-F2
M w/o w/I pellet purified
gB-F2-3
gB-F1-2

## Slide 2
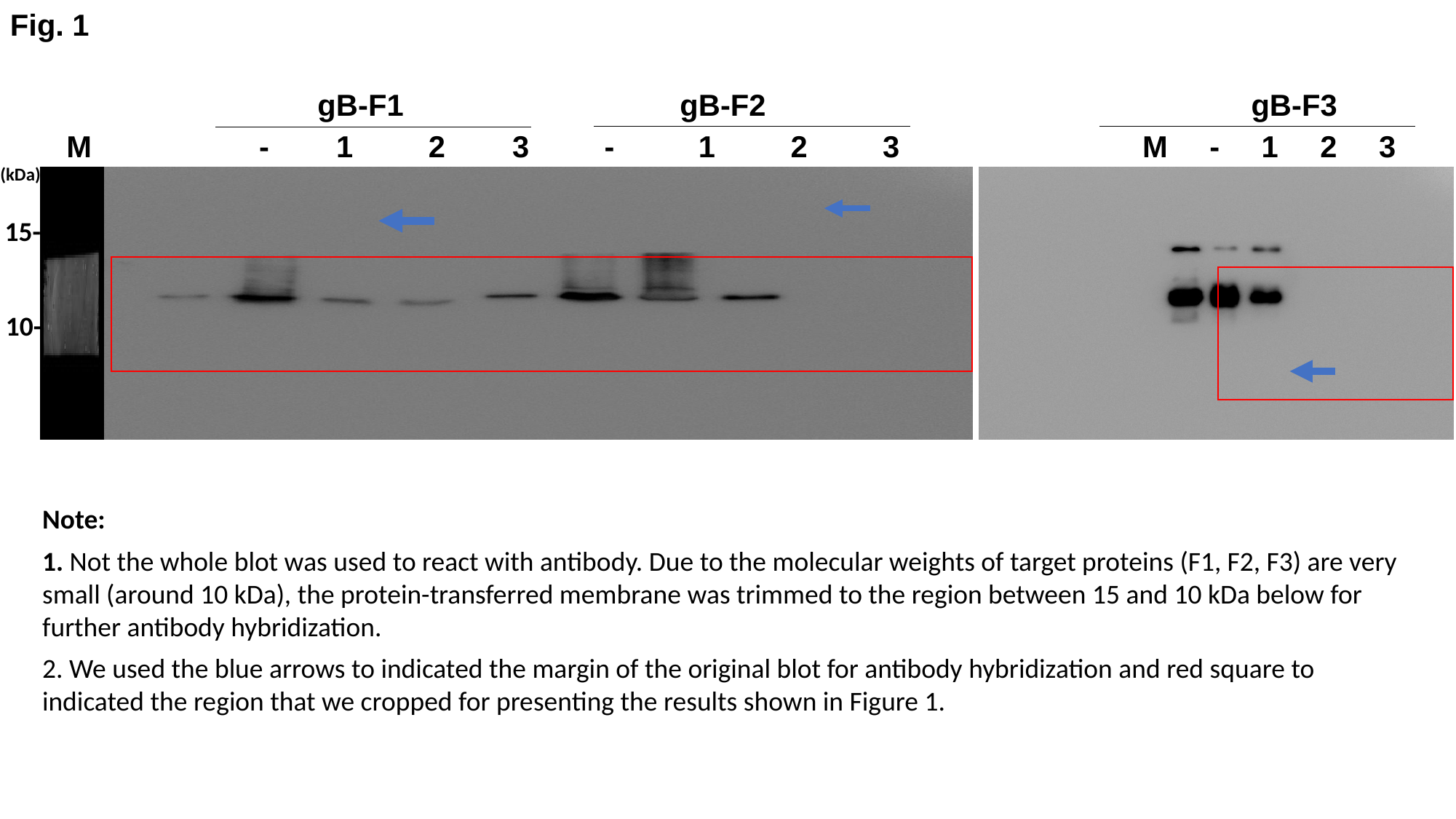

Fig. 1
gB-F1 gB-F2 gB-F3
M - 1 2 3 - 1 2 3 M - 1 2 3
(kDa)
15-
10-
Note:
1. Not the whole blot was used to react with antibody. Due to the molecular weights of target proteins (F1, F2, F3) are very small (around 10 kDa), the protein-transferred membrane was trimmed to the region between 15 and 10 kDa below for further antibody hybridization.
2. We used the blue arrows to indicated the margin of the original blot for antibody hybridization and red square to indicated the region that we cropped for presenting the results shown in Figure 1.

## Slide 3
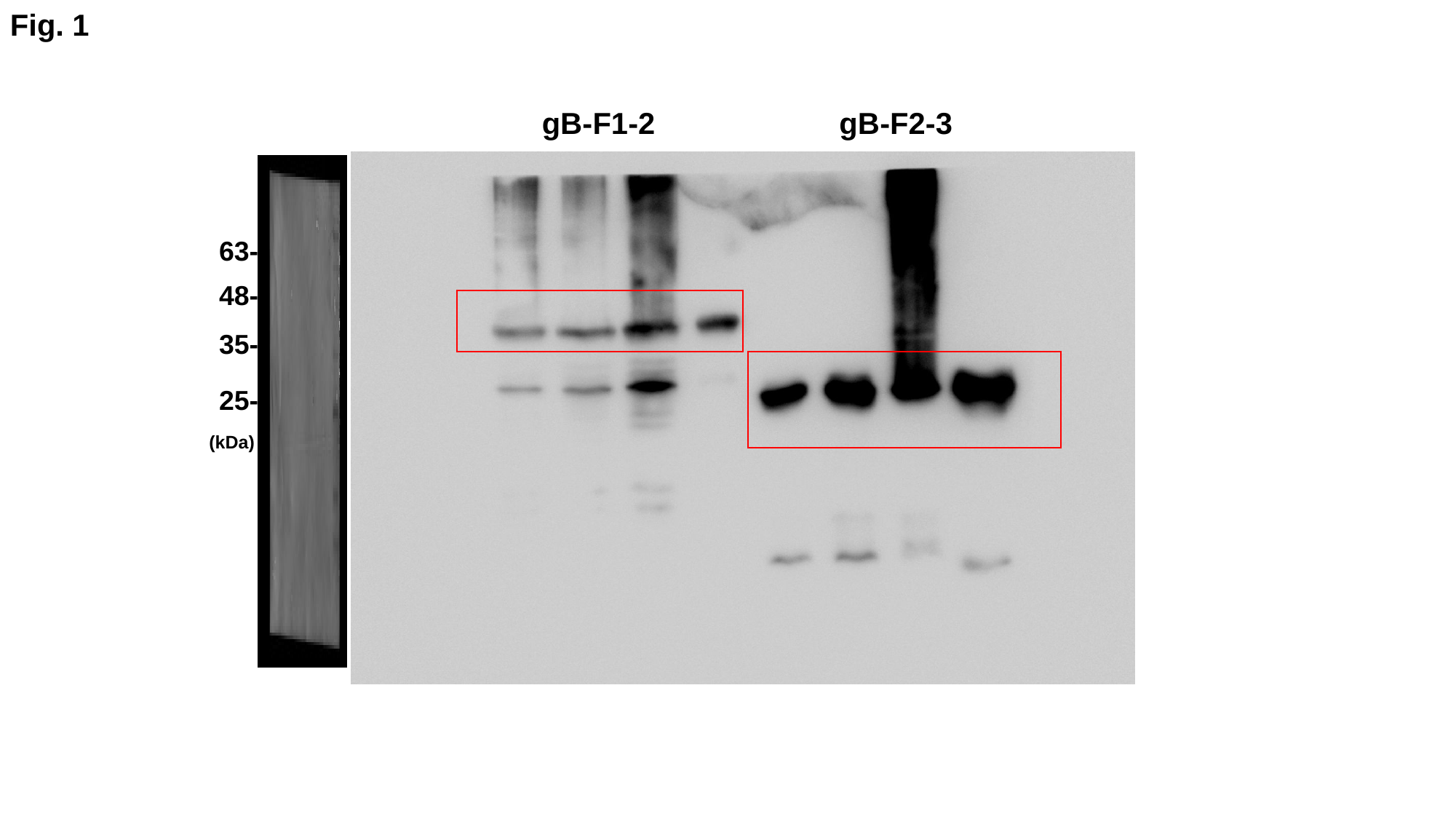

Fig. 1
gB-F1-2 gB-F2-3
63-
48-
35-
25-
(kDa)

## Slide 4
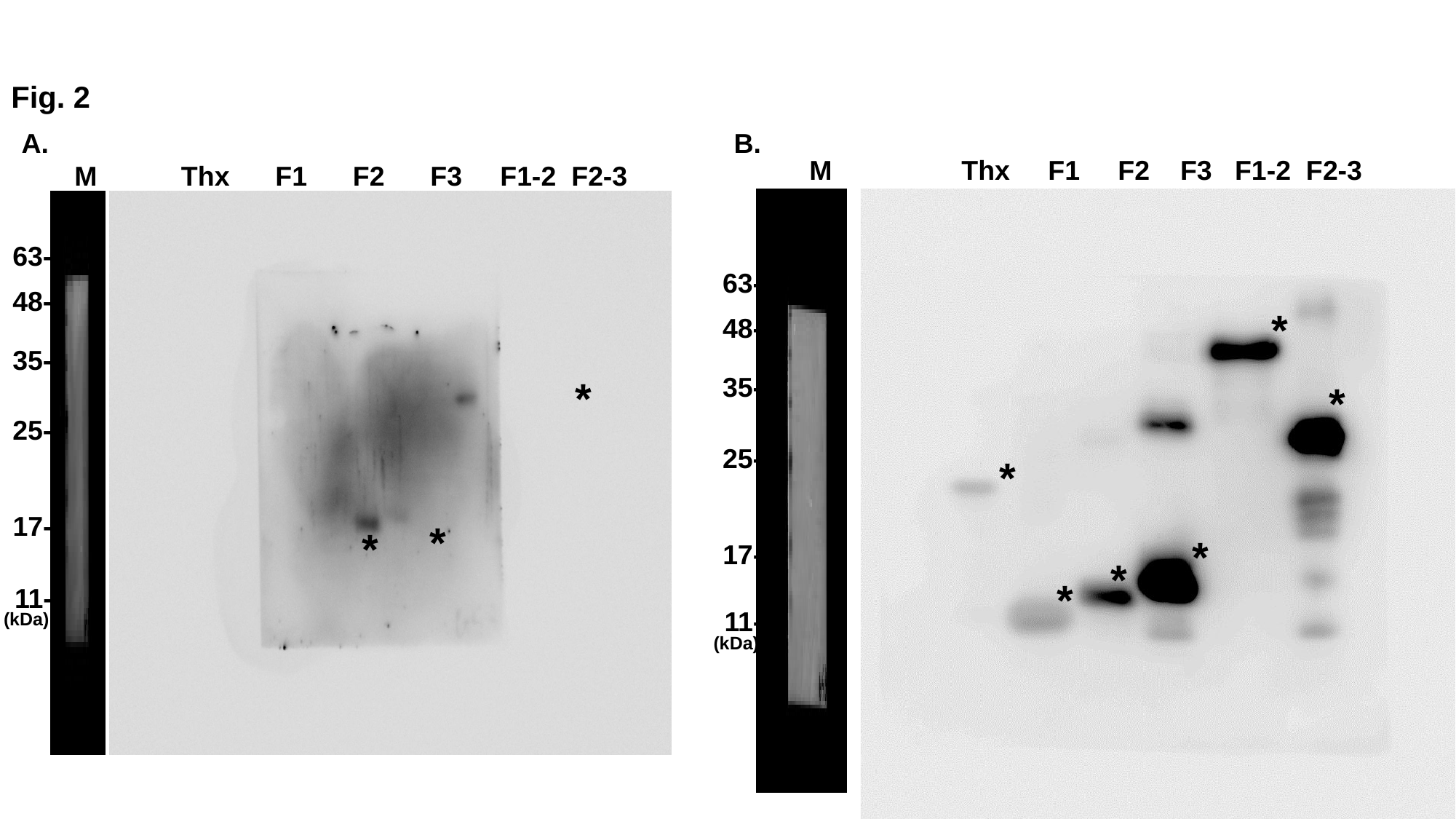

Fig. 2
A. B.
M Thx F1 F2 F3 F1-2 F2-3
M Thx F1 F2 F3 F1-2 F2-3
63-
63-
48-
*
48-
35-
35-
*
*
25-
25-
*
17-
*
*
*
17-
*
*
11-
11-
(kDa)
(kDa)

## Slide 5
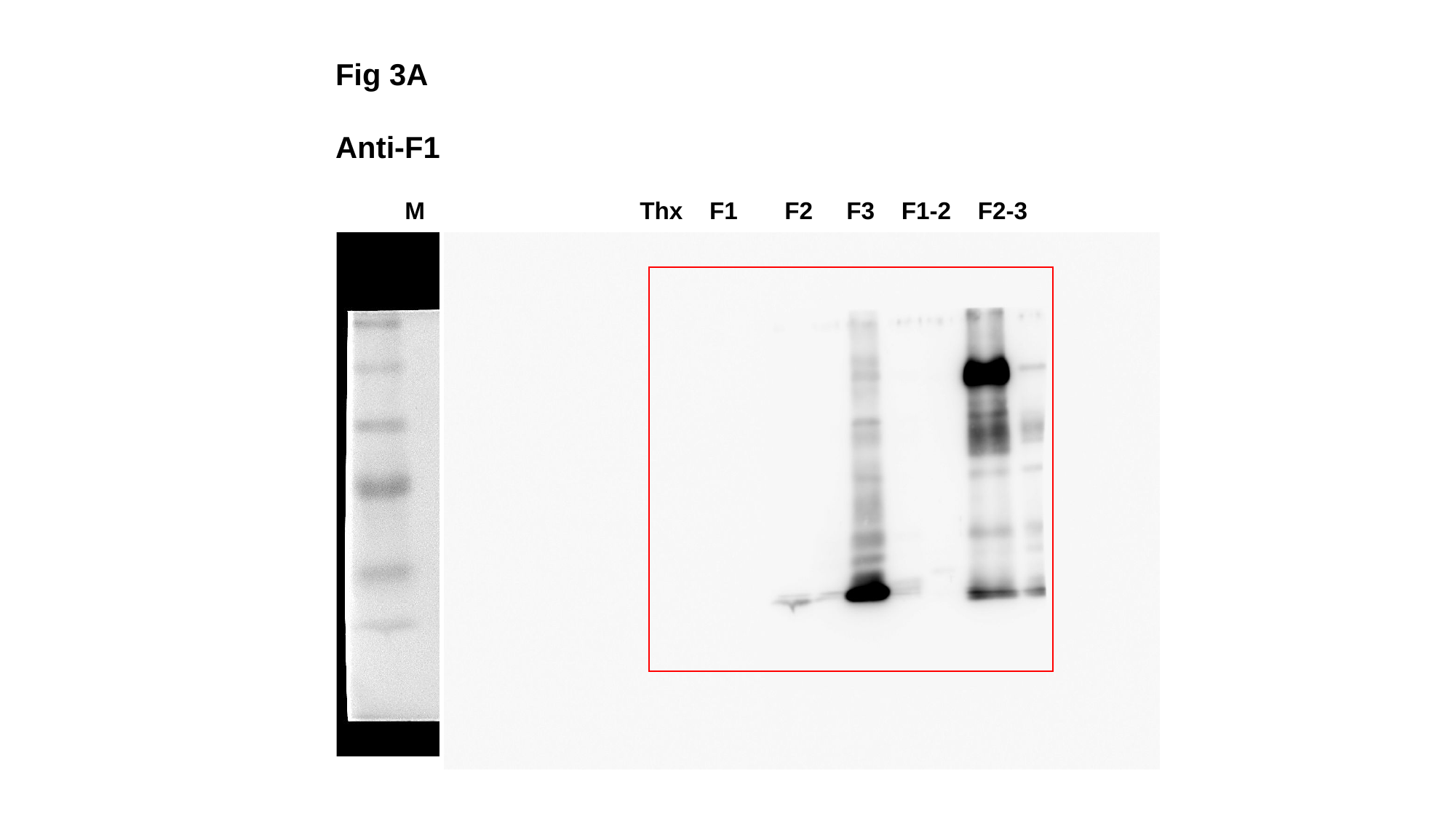

Fig 3A
Anti-F1
M Thx F1 F2 F3 F1-2 F2-3

## Slide 6
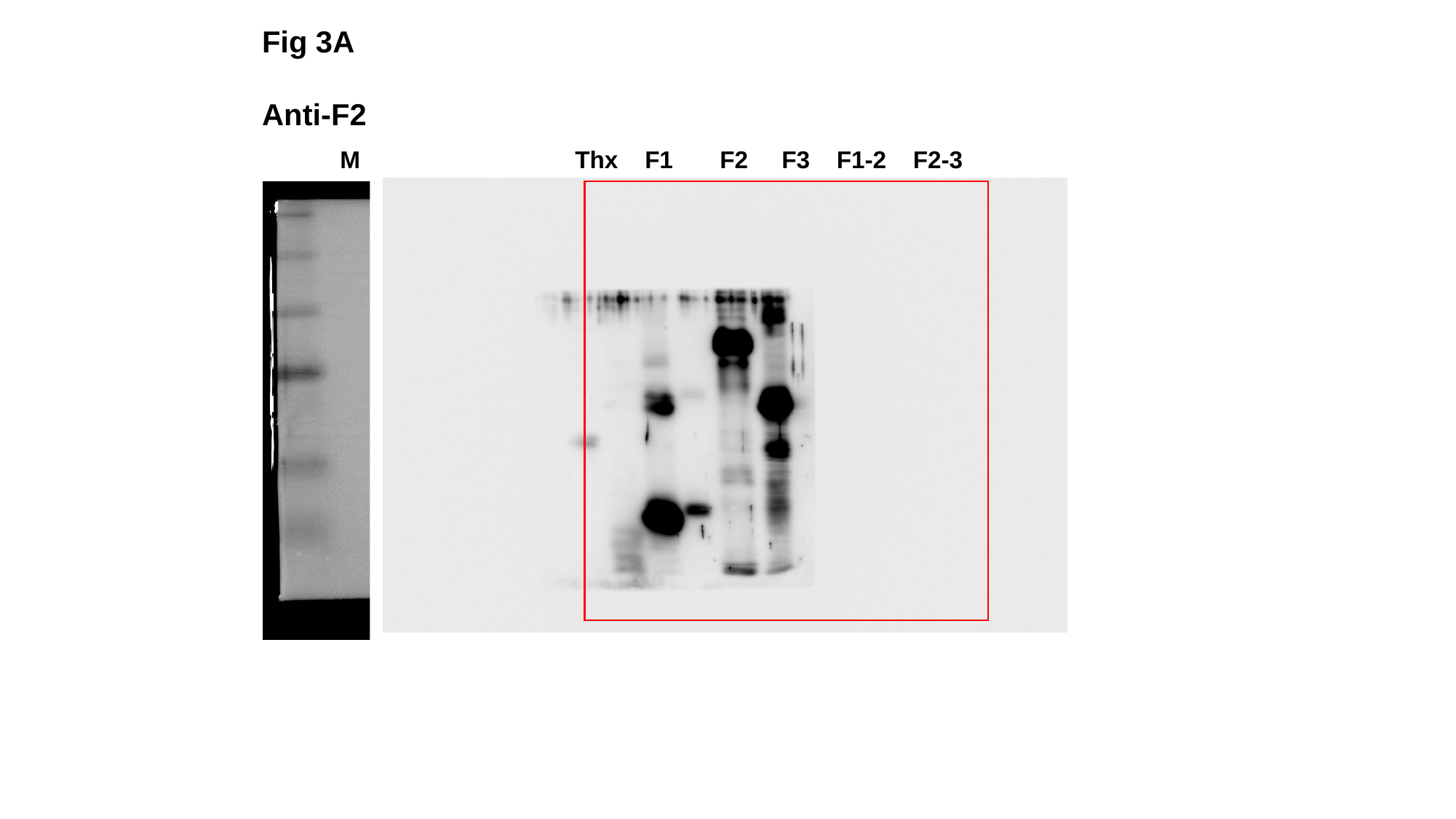

Fig 3A
Anti-F2
M Thx F1 F2 F3 F1-2 F2-3

## Slide 7
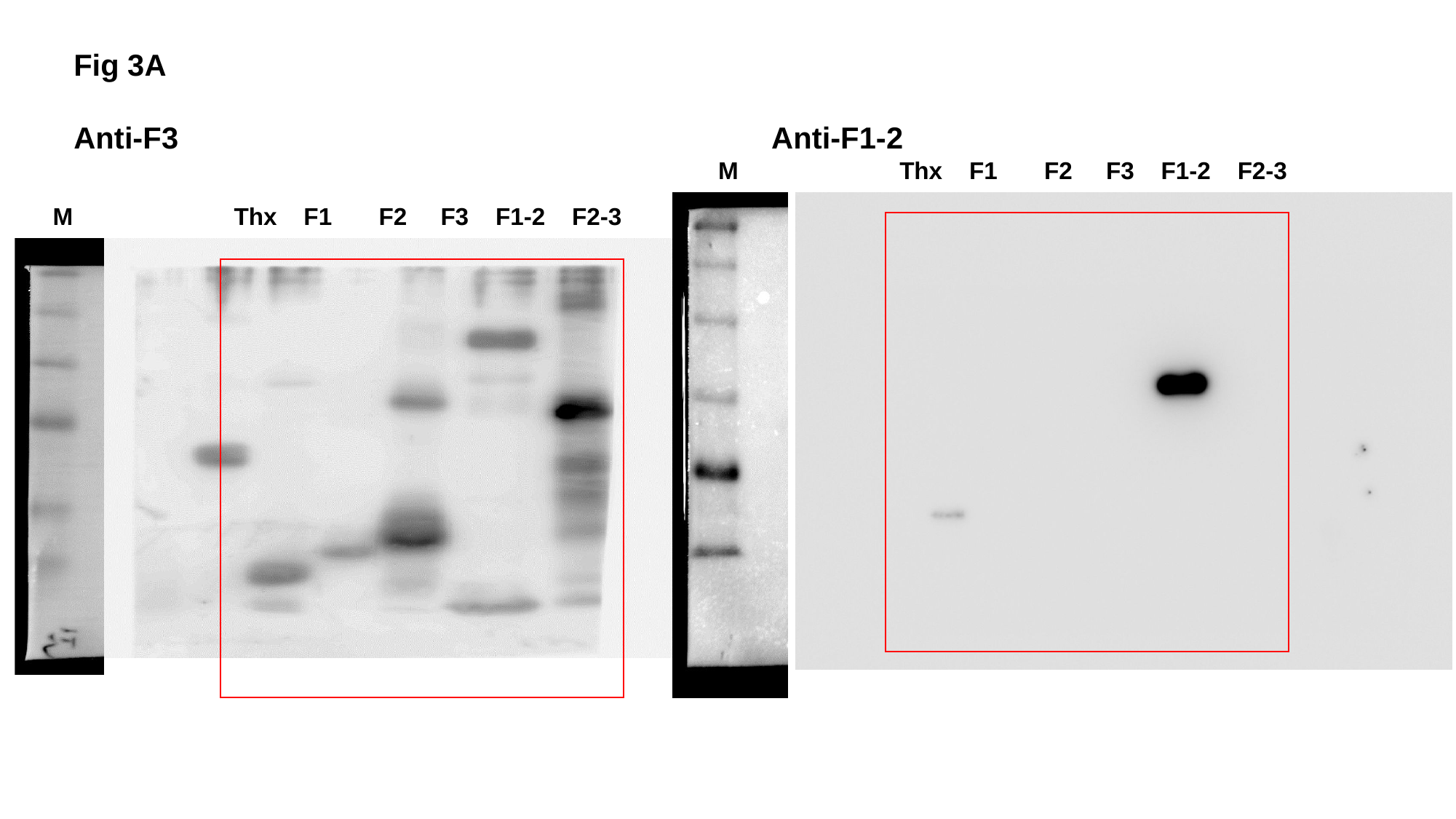

Fig 3A
Anti-F3 Anti-F1-2
M Thx F1 F2 F3 F1-2 F2-3
M Thx F1 F2 F3 F1-2 F2-3

## Slide 8
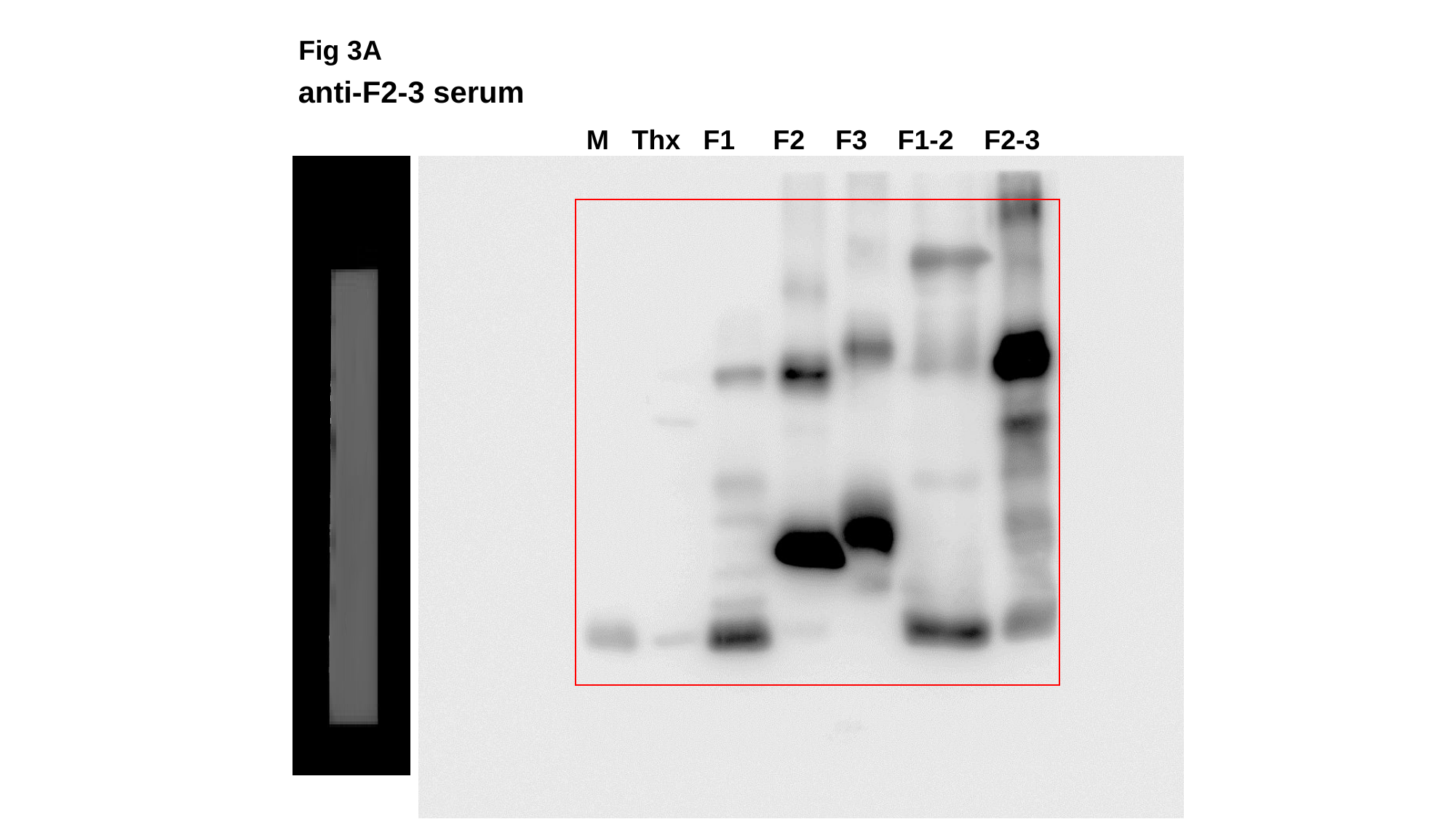

Fig 3A
anti-F2-3 serum
M Thx F1 F2 F3 F1-2 F2-3

## Slide 9
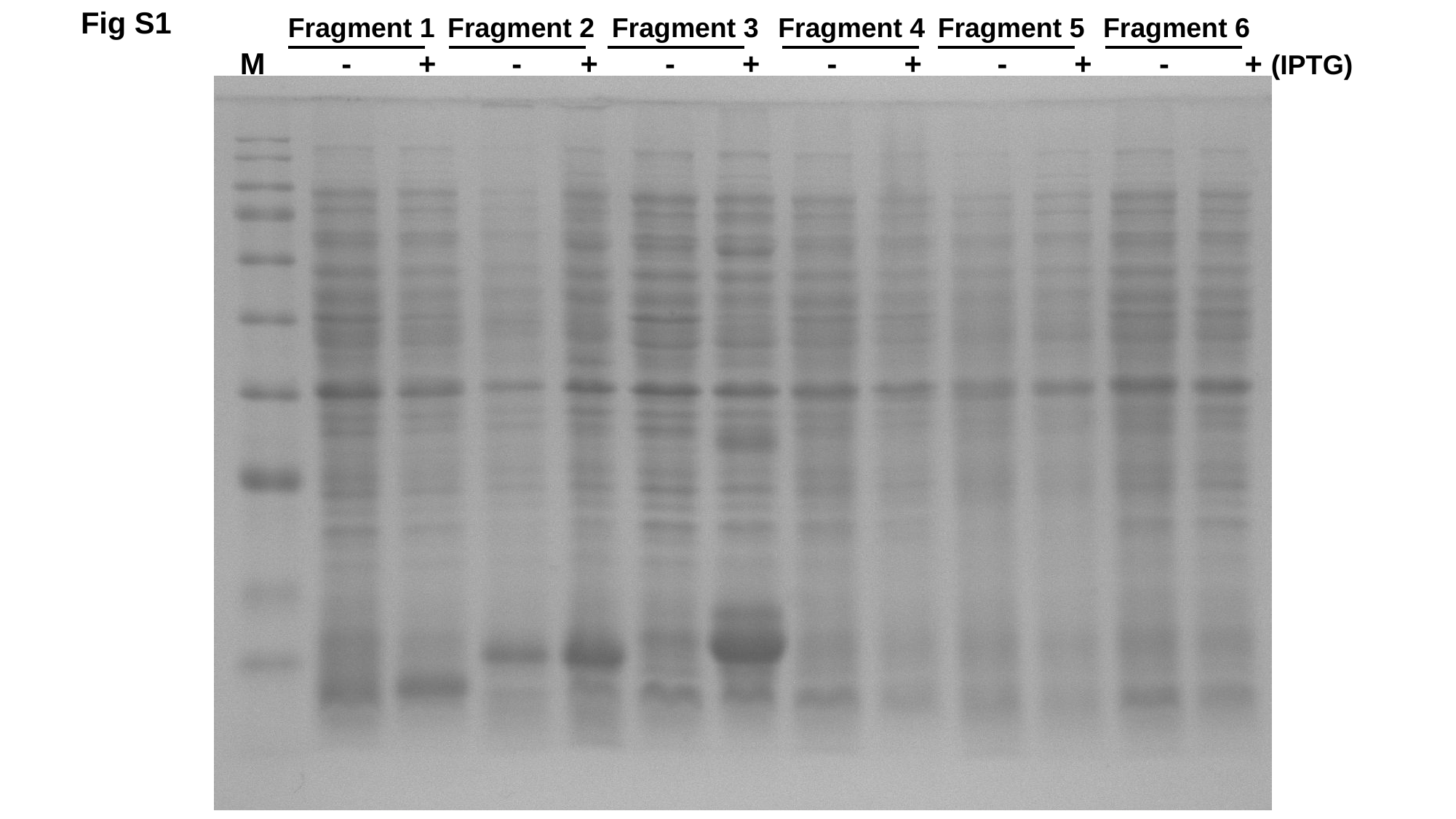

Fig S1
Fragment 1
Fragment 2
Fragment 3
Fragment 4
Fragment 6
Fragment 5
M - + - + - + - + - + - + (IPTG)
