# Supplementary material for: Preparation of recombinant glycoprotein B (gB) of Chelonid herpesvirus 5 (ChHV5) for antibody production and its application for infection detection in sea turtles
Source: Sci Rep. 2022 Jun 30;12:11022. doi: 10.1038/s41598-022-15281-9 (PMC9246996; doi:10.1038/s41598-022-15281-9)
Supplement: Supplementary file 3 — Supplementary Information 3. [file 41598_2022_15281_MOESM3_ESM.docx]

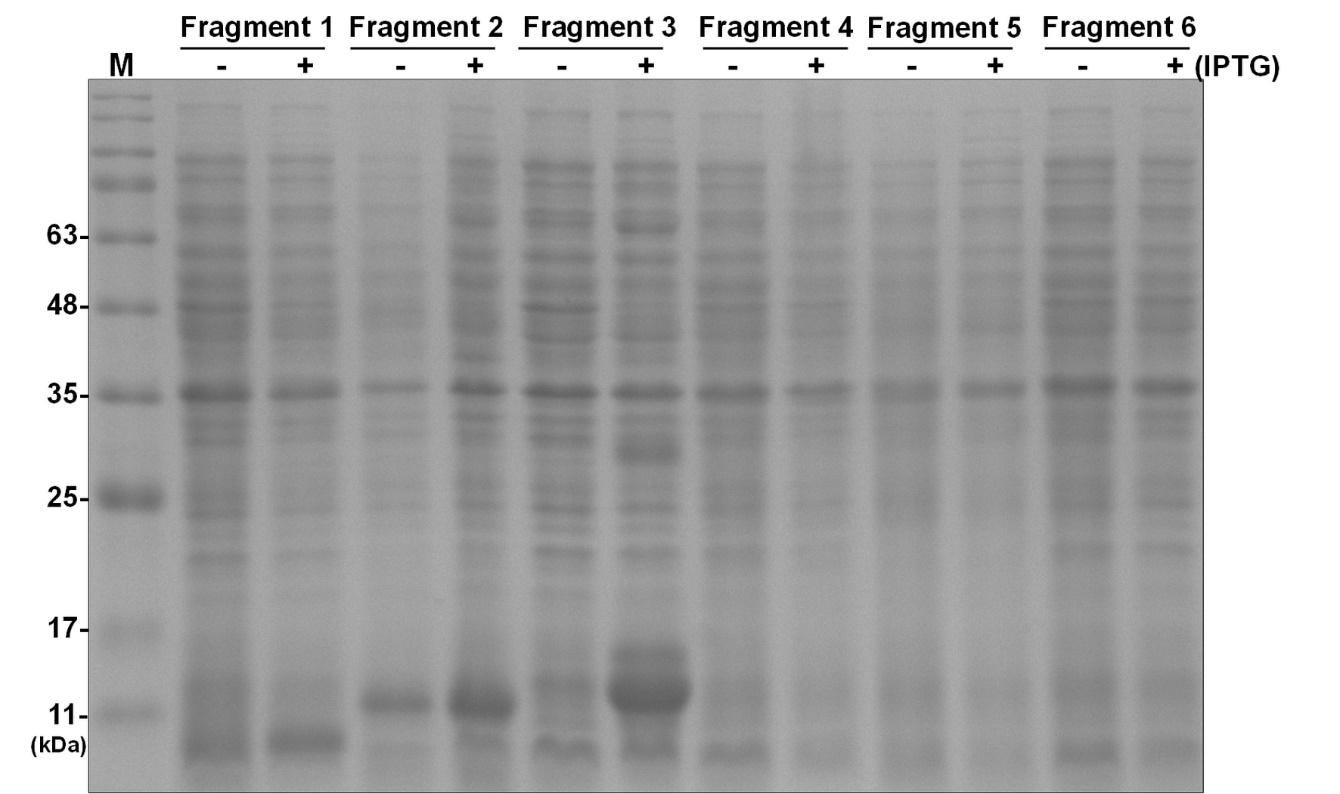


**Supplementary Figure S1. Expression of recombinant partial gB proteins.** Six antigenic fragments that cover entire region of gB protein, labelled as Fragment 1-Fragment 6, were expressed in *E. coli* under IPTG treatment. Lane M is side marker Line; - indicates mock control lysate without IPTG induction. Only fragment 1, 2, 3 were successfully over-expressed.
